# Decline in unmet needs for cataract surgery among the ageing population in India: findings from LASI, wave-1

**DOI:** 10.3389/frhs.2024.1365485

**Published:** 2024-03-19

**Authors:** Sasmita Das, Abhinav Sinha, Srikanta Kanungo, Sanghamitra Pati

**Affiliations:** Division of Public Health, ICMR-Regional Medical Research Centre, Bhubaneswar, India

**Keywords:** cataract, surgery, older adults, LASI, unmet needs

## Abstract

**Introduction:**

Cataracts are the leading cause of blindness among older people, but they can be treated with corrective surgery. India boasts the oldest blindness control programme in the world. We aimed to assess the prevalence of cataract surgery, and we compared the determinants of undergoing cataract surgery and identified the unmet needs for cataract surgery among older adults in India.

**Methods:**

We included 52,380 individuals aged ≥50 years from the Longitudinal Ageing Study in India, wave-1. The primary outcome measures of our study were the prevalence of cataract surgery and the unmet need for cataract surgery. Multivariate analysis was executed to investigate the association between socio-demographic variables and outcomes, expressing the results as adjusted odds ratios with 95% confidence intervals (CIs).

**Results:**

The overall prevalence of cataracts was 14.85%. The coverage of cataract surgery was 76.95%, with 23% having unmet needs for cataract surgery. Notably, cataract surgery coverage was higher at 78.30% (95% CI: 76.88–79.48) among participants aged 66–80 years, while the percentage of those who did not undergo cataract surgery was higher at 24.62% (95% CI: 23.09–26.20) among participants aged 50–60 years. The most deprived group had a higher odds ratio [adjusted odds ratio: 1.20 (95% CI: 1.00–1.44)] (*p* < 0.05) of having unmet needs for cataract surgery.

**Conclusions:**

There is a considerable burden of age-related cataracts in India. While the coverage of cataract surgery is high, the unmet need for cataract surgery cannot be overlooked. The existing blindness control programme has contributed significantly to increasing the coverage of cataract surgery, but it still needs to be strengthened, especially to reach the most deprived sections of society.

## Introduction

Vision is among the most vital senses of humans, which utilizes over 30% of the brain’s capacity for processing information (1). Consequently, the fear of vision loss ranks the highest among all possible disabilities (1, 2). Cataracts are the leading cause of curable blindness worldwide (3). According to the World Health Organization (WHO), as of 20121, there are an estimated 2.2 billion individuals living with near or distant visual impairment (4). Approximately 1 billion people suffer from moderate or severe distance visual impairment, with almost 94 million diagnosed with cataracts (4). Cataracts were responsible for 45% of global blindness in 2020 (4–6). In addition, cataracts are recognized as the second most common cause of moderate and severe vision impairment (MSVI) (7).

Cataracts may be congenital, secondary to trauma, or drug-induced, but the most common presentation is as age-related disease (3). Age-associated cataracts are mainly caused by the opacification of the lens due to oxidative damage (3). This form of blindness is associated with considerable disability and excess mortality, hampering the social and economic growth of the individual (8). Surgery is the most successful and least complicated treatment, leading to direct improvements in visual acuity and activities of daily living (ADL) along with decreased cataract-related morbidity and mortality (3). However, the distribution of cataracts is grossly uneven in low- and middle-income countries (LMICs) compared to high-income countries (HICs), with LMICs contributing 90% and HICs contributing 50% of all blindness (1, 8). LMICs contribute 90% of disability-adjusted life years (DALYs) due to cataracts, thus creating gross inequalities in the burden of cataracts (8).

More than 90% of individuals with visual impairment caused by cataracts live in LMICs (9–13). The prevalence of cataracts is increasing among LMICs, attributed to their rapid ageing population (8). For instance, data from India's 2011 census indicated that the proportion of individuals aged 60 years and above was around 8.6% of the total population (14). This percentage has been consistently on the rise due to declining birth rates and advancements in healthcare, contributing to extended life expectancy. Projections suggest that this demographics will further increase to 19.5% of the total population, reaching approximately 319 million by 2050 (15). More than half of the people experiencing blindness due to cataracts remain unreported due to lack of medical facilities in the vicinity, higher cost, gender bias, lack of awareness about available treatments, and fear of surgery (15). Effects of cataracts are not only confined to visual deficit but also to functional and psychological disability, which significantly reduces health-related quality of life (HRQoL) (16). India was the first country to start a blindness control programme in the world with the National Programme for Control of Blindness and Visual Impairment in 1976. The programme aimed to reduce blindness and visual impairment through various interventions, including medical treatment, surgeries, and awareness campaigns. It is worth noting that the specific types of cataract surgeries performed under this programme include manual small incision cataract surgery (MSICS) and phacoemulsification. According to the National Blind Visual Impairment Survey 2019, the coverage for cataract surgery among blind individuals and visually impaired people was 93.2% and 74.0% in India, respectively (17). As India has one of the oldest blindness control programmes, it is pertinent to determine the factors associated with undergoing cataract surgery and compare them with those who did not opt for corrective surgery. The evidence generated can further help reduce the unmet needs for cataract surgery by informing existing programmes and guiding future policies. Hence, this study was conducted with the objective of estimating the prevalence of cataracts among adults aged ≥50 years in India using data from the Longitudinal Aging Study in India (LASI), wave-1. Further, we compared the determinants of undergoing cataract surgery and the unmet needs for cataract surgery among older adults in India.

## Methods

### Overview of data

This study used data from LASI, wave-1, conducted from April 2017 to December 2018. LASI is a nationally representative study conducted in collaboration with the Harvard T.S. Chan School of Public Health, the International Institute for Population Sciences (IIPS), and the University of Southern California under the Ministry of Health and Family Welfare, Government of India. This study was implemented to understand the social and health challenges faced by older adults aged ≥45 years in India. Data were collected from all the states and union territories except Sikkim. Detailed information on LASI can be obtained from the website of the International Institute of Population Sciences, Mumbai (18).

### Sample size and participants

A total of 72,250 individuals aged ≥45 years and their spouses (irrespective of age) were surveyed by LASI. We excluded participants aged <50 years in the purview of our objective. The final sample size comprised 52,380 individuals aged ≥50 years, who were included in the present study.

### Independent variables

The variables for the present study were derived from the individual survey schedule of LASI. We used variables from the socio-demographic section, such as age, sex, residence, caste, education, occupation, marital status, wealth index, and regions of India. Age was classified into three groups: 50–65, 66–80, and ≥80 years. The sex of the participants was reported as male or female based on the observation. The residence was classified as rural and urban. The caste of participants was categorized into four groups, i.e., scheduled caste, scheduled tribe, other backward classes, and “others,” which included merging responses such as “none of them” and “no caste/tribe.” Education status was grouped as follows: no formal education, primary (less than primary, primary. and middle school), secondary (secondary and higher secondary), and higher (diploma, graduate, postgraduate, and professional degree). Occupation was stratified into three categories: never worked, currently working, and currently not working; this classification was based on questions “Have you ever worked for at least 3 months during your lifetime?” and “Are you currently working?”. Marital status was classified into two groups: “living with a partner” for those who responded being currently married or in a live-in relationship, while those who indicated being “widowed, divorced, separated, deserted, or never married” were grouped as “living without a partner.” The wealth index was grouped as most deprived (2–4), and the most affluent based on the monthly per capita expenditure (MPCE) (19). The region was categorized into six zones: east, west, south, north, northeast, and central.

### Outcome variables

**Figure 1:**
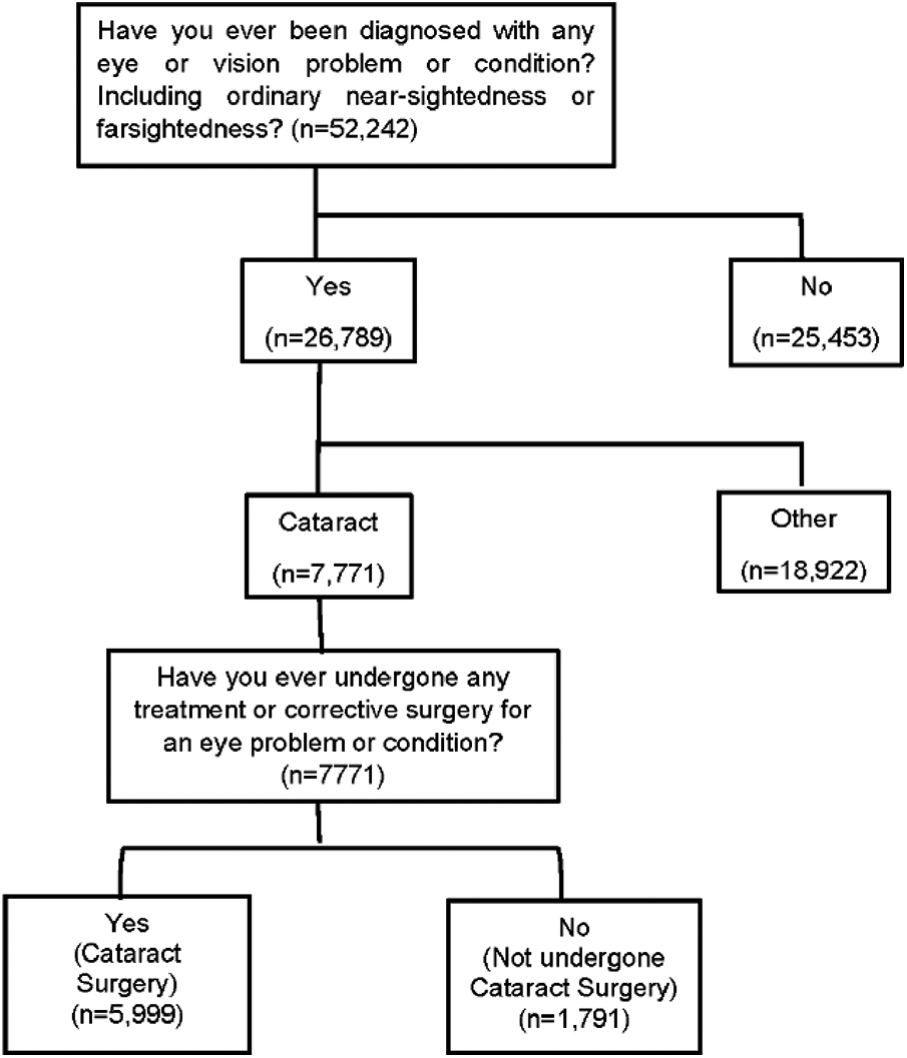
Selection of study participants.

The primary outcome measures of the study were the participants who had cataract surgery and those who did not. In the LASI individual questionnaire, the participants were asked, “Have you ever been diagnosed with any eye or vision problem or condition, including ordinary near sightedness or farsightedness?”; those who responded “yes” were further asked, “With which problem or condition were you diagnosed?”. Participants who self-reported “cataract” were considered to have the condition. They were then asked, “Have you ever undergone any treatment or corrective surgery for an eye problem or condition?”; those who responded “yes” and also mentioned the type of surgery were considered as having undergone surgery for cataracts. Those who responded “no” were considered as not having undergone cataract surgery (Figure 1).

### Statistical analysis

Data were analyzed using STATA version 16.0 (STATA Corp., TX, USA). Mean and standard deviation were used to report continuous variables such as age. Frequency and proportion (%) were tabulated for all socio-demographic variables alongside the outcome variable, i.e., cataract surgery. All weighted proportions were reported along with 95% confidence intervals (CIs) as a measure of uncertainty. We used binary logistic regression to assess the strength of association (odds ratio, OR) between various socio-demographic characteristics and the outcome variables. The variables with *p*-values less than 0.05 were considered significant and further included in the multivariable logistic regression model. This association was expressed as an adjusted odds ratio (AOR) with a 95% CI. Survey weights were considered in the analysis to compensate for complex survey designs. The variance inflation factor (VIF) was calculated to observe multicollinearity in the regression models.

### Ethical considerations

Because this study is a secondary analysis of data collected from LASI, it does not require any ethical approval. The original LASI study obtained ethics clearance from IIPS, Mumbai and the Indian Council of Medical Research, New Delhi. Written informed consent was obtained from all participants prior to their participation in the original LASI study.

## Results

The average age of the participants was 63.4 ± 9.57 years, ranging from 50 to 116 years. Most participants (63.16%) belonged to the 50–65 year category. Almost half of the participants (53.29%) were women. Almost two-thirds of the population resided in rural areas. The detailed socio-demographic characteristics of the study population are presented in Table 1. The gender-wise stratified socio-demographic characteristics of the study population are presented in Supplementary Table S1.

**Table 1:** Socio-demographic characteristics of the study participants.

Attributes	Categories	*n*	%
Age group (years) (*n* = 52,380)	50–65	33,084	63.16
	66–80	16,626	31.74
	>80	2,670	5.10
Gender (*n* = 52,380)	Male	24,465	46.71
	Female	27,915	53.29
Residence (*n* = 52,380)	Rural	36,172	69.06
	Urban	16,208	30.94
Caste (*n* = 51,929)	Scheduled caste	9,888	19.04
	Scheduled tribe	4,454	8.58
	Other backward class	23,751	45.74
	Others	13,836	26.64
Education (*n* = 52,379)	No formal education	27,776	53.03
	Primary complete	11,887	22.70
	Secondary complete	8,052	15.37
	Higher	4,664	8.90
Working status (*n* = 52,365)	Never worked	13,618	26.01
	Currently working	21,927	41.87
	Currently not working	16,820	32.12
Partner (*n* = 52,380)	With a partner	37,001	70.64
	Without a partner	15,379	29.36
Wealth index (*n* = 52,380)	Most deprived	11,163	21.31
	2	11,223	21.43
	3	10,749	20.52
	4	10,042	19.17
	Most affluent	9,203	17.57
Region (*n* = 52,380)	East	12,227	23.34
	West	8,726	16.66
	North	3,871	7.39
	South	12,520	23.90
	Northeast	1,685	3.22
	Central	13,351	25.49

### Prevalence of cataracts and cataract surgery

The overall prevalence of cataracts among the study participants was 14.85%. Among those with cataracts, 76.95% had undergone corrective surgery, while 23% had not undergone surgery.

Table 2 presents the distribution of participants who underwent cataract surgery and those who did not across various socio-demographic characteristics. The coverage of cataract surgery was higher (78.30%, 95% CI: 76.88–79.48) among participants aged 66–80 years, whereas younger participants aged 50–65 years had a higher proportion (24.62%, 95% CI: 23.09–26.20) of not undergoing corrective surgery. A higher (77.54%, 95% CI: 76.31–78.76) number of women had cataract surgery compared to men, while cataract surgery was less preferred (23.59%, 95% CI: 22.13–25.09) by men. Participants residing in rural areas had a higher coverage of (77.96%, 95% CI: 76.76–79.11) cataract surgery, whereas those in urban areas had a higher unmet need for cataract surgery. The highest unmet need for cataract surgery was observed among participants with the highest level of education (26.35%, 95% CI: 22.69–30.13), while respondents with no formal education (78.36%, 95% CI: 77.05–79.59) had undergone more cataract surgeries. The coverage for cataract surgery decreased with an increase in wealth, i.e., the most deprived group (82.88%, 95% CI: 80.87–84.62) had the highest number of cataract surgeries, while this group had the lowest (17.12%, 95% CI: 15.25–18.99) unmet need of cataract surgery. The prevalence of cataract surgery was higher in the central region (84.11%, 95% CI: 82.42–85.72), while the west (29.47%, 95% CI: 27.37–31.59) region had more unmet need for cataract surgery. The gender-wise stratified distribution of participants who underwent cataract surgery across various socio-demographic characteristics is presented in Supplementary Table S2.

**Table 2:** Distribution of participants who had cataract surgery and those who did not across various socio-demographic attributes.

Attributes	Categories	Cataract surgery	No cataract surgery
		*n* (%) (95% CI)	*n* (%) (95% CI)
Age groups (years)	50–65	2,265 (75.38) (73.79–76.90)	740 (24.62) (23.09–26.20)
	66–80	3,090 (78.20) (76.88–79.48)	861 (21.80) (20.51–23.11)
	>80	633 (77.81) (74.74–80.57)	181 (22.19) (19.42–25.25)
Gender	Male	2,481 (76.41) (74.90–77.86)	766 (23.59) (22.13–25.09)
	Female	3,508 (77.54) (76.31–78.76)	1,016 (22.46) (21.25–23.70)
Residence	Rural	3,794 (77.96) (76.76–79.11)	1,073 (22.04) (20.88–23.23)
	Urban	2,194 (75.59) (73.97–77.13)	708 (24.41) (22.83–25.99)
Caste	Scheduled caste	1,140 (78.61) (74.41–80.70)	310 (21.39) (19.29–23.58)
	Scheduled tribe	286 (76.95) (72.25–81.07)	85 (23.05) (18.92–27.74)
	Other backward class	2,799 (79.00) (77.62–80.33)	744 (21) (19.66–22.37)
	Others	1,723 (73.63) (71.79–75.40)	617 (26.37) (24.59–28.20)
Education	No formal education	3,238 (78.36) (77.05–79.59)	894 (21.64) (20.38–22.91)
	Primary complete	1,488 (76.19) (74.23–78.06)	465 (23.81) (21.92–25.74)
	Secondary complete	847 (75.58) (72.93–78.04)	274 (24.42) (21.95–27.06)
	Higher	414 (73.65) (69.68–77.13)	148 (26.35) (22.69–30.13)
Working status	Never worked	1,834 (79.27) (77.54–80.89)	479 (20.70) (19.06–22.40)
	Currently working	1,432 (73.78) (71.75–75.72)	509 (26.22) (24.27–28.24)
	Currently not working	2,720 (77.43) (76.02–78.82)	792 (22.55) (21.11–23.97)
Partner	With a partner	3,463 (76.43) (75.14–77.64)	1,068 (23.57) (22.33–24.82)
	Without a partner	2,525 (77.97) (76.51–79.39)	713 (22.03) (20.60–23.48)
Wealth index	Most deprived	1,330 (82.88) (80.87–84.62)	274 (17.12) (15.25–18.99)
	2	1,324 (77.39) (75.32–79.34)	387 (22.61) (20.65–24.67)
	3	1,169 (74.72) (72.41–76.78)	395 (25.28) (23.08–27.45)
	4	1,197 (76.17) (73.95–78.23)	374 (23.83) (21.70–25.97)
	Most affluent	966 (73.44) (70.92–75.77)	349 (26.56) (24.15–28.99)
Region	East	1,349 (79.93) (77.92–81.80)	339 (20.07) (18.19–22.07)
	West	1,295 (70.53) (68.35–72.57)	541 (29.47) (27.37–31.59)
	North	317 (71.12) (66.78–75.40)	128 (28.88) (24.59–33.21)
	South	1,270 (74.41) (72.25–76.45)	436 (25.59) (23.48–27.68)
	Northeast	123 (81.93) (74.90–87.79)	27 (18.07) (12.20–25.09)
	Central	1,632 (84.11) (82.42–85.72)	308 (15.89) (14.27–17.57)

Table 3 presents the association of various socio-demographic characteristics with outcome variables, i.e., underwent cataract surgery and did not undergo cataract surgery. The univariable association showed that other caste (OR: 1.31, 1.03–1.67) and region were associated with having cataract surgery, whereas being in the most deprived group (OR: 1.42, 1.08–1.85) had a significant association with not undergoing cataract surgery. The multivariable regression model revealed that west (AOR: 1.70, 1.31–2.20), north (AOR: 1.54, 1.18–2.01), and south (AOR: 1.54, 1.17–2.03) regions were significantly associated with having cataract surgery. The determinant of not undergoing cataract surgery was being from the most deprived group (AOR: 1.35, 1.02–1.78). However, we did not find any association between different age groups, gender, residence, education, working status, and partner status in both univariable and multivariable analyses. We observed a VIF of 1.3, indicating that there was no multicollinearity in the existing regression model. The detailed VIF value for each of the variables is mentioned in Supplementary Table S3.

**Table 3:** Association between various socio-demographic attributes and cataract surgery done and not done.

Attributes	Categories	Cataract surgery			
		Yes		No	
		OR (95% CI)	AOR (95% CI)	OR (95% CI)	AOR (95% CI)
Age (years)	50–65	Reference			
	66–80	0.85 (0.70–1.03)	0.88 (0.72–1.08)	1.17 (0.96–1.41)	1.12 (0.92–1.35)
	81 and above	0.87 (0.63–1.20)	0.65 (0.65–1.30)	1.14 (0.83–1.57)	1.08 (0.76–1.53)
Gender	Female	Reference			
	Male	1.06 (0.88–1.27)	0.99 (0.77–1.29)	0.93 (0.78–1.12)	1.00 (0.77–1.29)
Residence	Rural	0.87 (0.72–1.06)	1.00 (0.77–1.29)	1.14 (0.93–1.38)	0.99 (0.77–1.28)
	Urban	Reference			
Caste	Scheduled caste	Reference			
	Scheduled tribe	1.10 (0.74–1.62)	1.08 (0.68–1.53)	0.90 (0.61–1.34)	0.97 (0.65–1.45)
	Other backward class	0.97 (0.76–1.2)	0.87 (0.68–1.12)	1.02 (0.79–1.31)	1.14 (0.88–1.46)
	others	1.31 (1.03–1.67)	1.23 (0.94–1.59)	0.75 (0.59–0.96)	0.81 (0.62–1.05)
Education	No formal education	0.77 (0.56–1.05)	0.91 (0.61–1.35)	1.29 (0.95–1.76)	1.09 (0.73–1.63)
	Primary completed	0.87 (0.63–1.20)	0.89 (0.62–1.26)	1.14 (0.83–1.57)	1.12 (0.79–1.58)
	Secondary completed	0.90 (0.64–1.26)	0.88 (0.62–1.25)	1.10 (0.78–1.55)	1.13 (0.79–1.60)
	Higher	Reference			
Working status	Currently not working	Reference			
	Never worked	0.89 (0.73–1.09)	0.90 (0.70–1.16)	1.11 (0.91–1.36)	1.10 (0.85–1.42)
	Currently working	1.21 (0.96–1.54)	1.23 (0.96–1.59)	0.82 (0.64–1.03)	0.80 (0.62–1.04)
Partner	With partner	1.09 (0.91–1.30)	1.02 (0.83–1.25)	0.91 (0.76–1.09)	0.97 (0.79–1.19)
	Without partner	Reference			
Wealth index	Most deprived	0.70 (0.54–0.92)	0.73 (0.56–0.97)	1.41 (1.08–1.85)	1.35 (1.02–1.78)
	2	Reference			
	3	1.15 (0.87–1.5)	1.16 (0.87–1.56)	0.86 (0.64–1.14)	0.85 (0.64–1.14)
	4	1.07 (0.79–1.43)	1.05 (0.78–1.43)	0.93 (0.69–1.25)	0.94 (0.69–1.27)
	Most affluent	1.23 (0.95–1.60)	1.19 (0.90–1.57)	0.80 (0.62–1.04)	0.83 (0.63–1.10)
Region	East	Reference			
	West	1.66 (1.29–2.13)	1.70 (1.31–2.20)	0.60 (0.46–0.77)	0.58 (0.45–0.76)
	North	1.61 (1.24–2.09)	1.54 (1.18–2.01)	0.61 (0.47–0.80)	0.64 (0.49–0.84)
	South	1.36 (1.02–1.83)	1.54 (1.17–2.03)	0.73 (0.54–0.98)	0.64 (0.49–0.84)
	North-east	0.87 (0.60–1.28)	0.86 (0.58–1.27)	1.13 (0.77–1.66)	1.15 (0.48–1.70)
	Central	0.75 (0.56–0.99)	0.80 (0.60–1.06)	1.32 (1.00–1.75)	1.24 (0.94–1.64)

CI, confidence interval; OR, odds ratio.

## Discussion

People aged 50 years and above represent around 13% of the total Indian population (14). This study provides novel insights into the prevalence of cataracts, coverage of cataract surgery, and unmet needs among a nationally representative sample of participants aged 50 years and above, which is important for the programme. We observed a high coverage of cataract surgery and a lower unmet need among the study participants.

Age-related cataracts are the leading cause of visual impairment and blindness, requiring timely intervention in the form of surgery (19). Our analysis reveals that participants aged 66–80 years had a higher prevalence of cataract surgery, followed by respondents aged >80 years. Our findings are consistent with the results of a similar study conducted among a representative Finnish population, which showed a relatively higher coverage of cataract surgery with increasing age (20). The proportion of unmet surgical need, however, was greater (39.1%) in previous geriatric population studies carried out by Wasekar and Lavangare in Mumbai and Vimalraj et al. in Kerala (21, 22). Moreover, shorter operating times, availability of more efficient anaesthesia, and a trend of daycare surgery have made cataract surgery a minor surgical procedure. Nonetheless, we observed that participants aged 50–65 years had the highest unmet needs for cataract surgery. A probable reason for this could be that people in this age group do not seek medical intervention until the disease progresses to an advanced stage. Although there were variations in the coverage of cataract surgery and unmet needs, no significant difference was observed between the age groups in both categories. This reflects the awareness among the masses, which has been a cumulative effect of the ongoing blindness control programme.

We observed that women had a higher coverage and lower unmet needs for cataract surgery. However, our findings contradict previous reports that show a lower coverage of cataract surgery among women (20–25). Nonetheless, our findings do not show a significant association between gender and outcome variables. Interestingly, no significant association was observed across urban and rural residents in accessing cataract surgery. In addition, contrary to prior studies, we found that rural residents had higher coverage and fewer unmet needs for cataract surgery than urban residents (26). However, multiple barriers contribute to urban–rural disparities in accessing healthcare facilities, including lack of financial resources, availability of healthcare setups, geographical location, unavailability of human resources, and civic amenities (15, 27). Still, a higher coverage indicates the strength of India's existing National Programme for Control of Blindness, which aims to prevent visual disability by providing free camps with free diagnostics and treatment services for cataract surgery. The various steps taken to reduce the burden of unmet need for cataract surgery include organization of mass surgery camps and awareness campaigns through various Information, Education, and Communication (IEC) activities in the community. In addition, the decentralization of government healthcare facilities, along with recent efforts to strengthen primary care by establishing Health and Wellness Centres, has helped in reducing this disparity (27). Similar findings were reported by Nathenetel et al. and Madaki et al. in Nigeria, where cataract surgery coverage showed a slight improvement (28, 29).

Individuals with no formal education and those who never worked had a higher coverage of cataract surgery. This further strengthens our notion that the existing programme has been successful in reaching each stratum of society. However, we observed a significant association between unmet needs for cataract surgery and the most deprived quintile. This is consistent with the findings of another study, which showed a higher risk of developing cataracts among the most deprived people (20). This might be due to a lack of awareness, inability to pay, or other socio-cultural factors, which further need to be explored. To achieve the global WHO target of increasing coverage of cataract surgery by 30% by 2030 and achieving universal health coverage, the most disadvantaged group must also have egalitarian access to healthcare facilities (25).

Participants belonging to the west, north, and south regions were significantly associated with having cataract surgery, while no significant association for unmet needs for cataract surgery was observed. This points towards replicating the best practices adopted by these regions in other regions to achieve zero blindness. In addition, socio-cultural factors of patients, their health systems, and awareness play an important role in this uptake, which needs to be explored further. Moreover, a probable reason for the central and north-eastern zones to have fewer cataract surgeries may be due to disparities in health infrastructure, geographic remoteness, and differences among the participants studied, which can also impede access challenges (31, 32). Moreover, health-seeking behaviour also varies between the regions, which could also be a probable reason for this difference (33). An Iranian study also found that cataract surgery coverage is low due to low cost and other resources (30).

### Implications for policy and practice

Our study shows a high coverage of corrective surgery for cataracts, reflecting the strength of our existing blindness control programme. However, to achieve the goals of VISION-2020, we need to focus on the most deprived strata of society by creating awareness among them regarding the importance of undergoing surgery. In addition, there is a need to build trust in publicly funded camps by assuring them of the quality of care during surgery and their safety. IEC activities and camps should aim for zero blindness. Middle-aged adults should be motivated to undergo surgery when advised. Regional differences should be addressed by adopting the best practices from regions or states that are performing well. This provides actionable guidance for shaping future programme strategies, emphasizing the importance of equitable access, awareness campaigns, early intervention, and replicating successful practices. Addressing regional disparities and focusing on the most deprived groups are keys to achieving the goals of initiatives like VISION-2020 and realizing universal health coverage.

### Strengths and limitations

A major strength of this study is the use of a large nationally representative sample, making it the first study to generate nationwide evidence on the coverage of cataract surgery. However, our study was limited by self-reported cataract diagnosis, which might have undermined the true prevalence, with answers potentially subject to recall bias. Also, cataract diagnosis alone as a measure of unmet needs might overstate the actual surgical necessity as many cataracts may not require surgery immediately. However, assessing the stage of cataracts was beyond the scope of this study as adequate, pertinent data were not available. Given the fact that some cataracts may not require surgery but can proceed to an advanced stage, in the absence of data, our operational definition of “unmet need” may be the best estimate, although future studies should employ more refined clinical criteria to better assess the urgency and appropriateness of surgical intervention, ensuring a more accurate depiction of true unmet need. In addition, our study was also limited by the lack of a clear definition of the type of cataract surgery, as relevant data were not available. Hence, this study encompasses cataract surgery as any type of cataract surgery, such as MSICS or phacoemulsification.

## Conclusion

Although there is a considerable burden of age-related cataracts in India, the coverage of cataract surgery is high. Nonetheless, the unmet needs for cataract surgery cannot be overlooked. The existing blindness control programme has contributed significantly to increasing the coverage of cataract surgery, but it still needs to be strengthened, especially to reach the most deprived sections of society. In addition, efforts to increase awareness should be continued, and regional differences should be managed by adopting the best practices of areas with higher coverage of corrective surgery.

## Data Availability

The datasets presented in this study can be found in online repositories. The names of the repository/repositories and accession number(s) can be found in https://g2aging.org/?section=overviews&amp;study=lasi.
