# Supplementary material for: Decline in unmet needs for cataract surgery among the ageing population in India: findings from LASI, wave-1
Source: Front Health Serv. 2024 Mar 19;4:1365485. doi: 10.3389/frhs.2024.1365485 (PMC10985141; doi:10.3389/frhs.2024.1365485)
Supplement: Supplementary file 1 [file Table1.docx]

| Supplementary Table S1: Socio-demographic characteristics of the study participants across gender | | | |
| --- | --- | --- | --- |
| Attributes | Categories | Male n (%) | Female n (%) |
| Age Group (Years)  n=52380 | 50-65 | 15055.6(45.5) | 18028(54.4) |
|  | 66-80 | 8168.8(49.1) | 8457.4(50.8) |
|  | >80 | 1240.5(46.4) | 1429.5(53.5) |
| Residence  n=52380 | Rural | 17056.3(47.1) | 19115.7(52.8) |
|  | Urban | 7408.6(45.7) | 8799.2(54.2) |
| Caste  n=51929 | Scheduled Caste | 4565.2 (46.17) | 5323.0(53.8) |
|  | Scheduled Tribe | 1995.4(44.8) | 2458.8(55.2) |
|  | Other Backward Class | 11266.9(47.4) | 12483.6(52.5) |
|  | others | 6467.3(46.7) | 7368.5(53.2) |
| Education  n=52379 | No formal education | 8745.4(31.4) | 19030.2(68.5) |
|  | Primary complete | 6923.2(58.2) | 4964.4(41.7) |
|  | Secondary complete | 5357.5(66.5) | 2694.2(33.4) |
|  | Higher | 3439.0(73.7) | 1224.6(26.2) |
| Working Status  n=52365 | Never worked | 778.5(5.7) | 12840(94.2) |
|  | Currently Working | 14531.9(66.2) | 7394.8(33.7) |
|  | Currently not Working | 9142.8(54.3) | 7676.7(45.6) |
| Partner  n=52380 | With Partner | 21079.9(56.9) | 15920.7(43) |
|  | Without Partner | 3385.0(22.0) | 11994.3(77.9) |
| Wealth Index  n=52380 | Most deprived | 5020.5(44.9) | 6142(55) |
|  | 2 | 5162.4(46) | 6060(54) |
|  | 3 | 5040(46.8) | 5709.3(53.1) |
|  | 4 | 4647.2(46.28) | 5394.7(53.7) |
|  | Most affluent | 44594.4(49.9) | 4608.5(50) |
| Region  n=52380 | East | 5847.5 (47.83) | 6379.2(52.17) |
|  | West | 3861.1(44.25) | 4864.8 (55.75) |
|  | North | 1801.7 (46.5) | 2068.7 (53.45) |
|  | South | 5702 (45.5) | 6817.5 (54.45) |
|  | North-east | 780.9 (46.3) | 904.2 (53.6) |
|  | Central | 6470 (48.4) | 6880.4 (51.53) |

| Supplementary Table S2: Gender wise distribution of participants who had and did not undergo cataract surgery across various socio-demographic attributes. CI: confidence interval | | | |
| --- | --- | --- | --- |
| Attributes | Categories | Male  n, %(95%CI) | Female  n, %(95%CI) |
| Age Group (Years) | 50-65 | 849,37.5(35.53-39.56) | 1413,62.4(60.38-64.42) |
|  | 66-80 | 1348,43.6(41.90-45.43) | 1737,56.32(54.56-58.9) |
|  | >80 | 280,44.3(40.38-48.27) | 352,55.32(51.72-59.61) |
| Residence | Rural | 1642,43.35(41.75-44.93) | 2147,56.6(55.06-58.24) |
|  | Urban | 835,38(36.07-40.18) | 1356,61.9(59.81-63.92) |
| Caste | Scheduled Caste | 486,42.8(40.14-45.98) | 650,57.1(54.28-60.11) |
|  | Scheduled Tribe | 104,36.5(30.78-42.2) | 181,63.42(57.41-68.88) |
|  | Other Backward Class | 1155,41.3(39.54-43.23) | 1635,58.6(56.72-60.41) |
|  | others | 711,41.3(39.04-43.75) | 1007,58.6(56.24-60.95) |
| Education | No formal education | 785,24.2(22.81-25.79) | 2449,75.7(74.23-77.21) |
|  | Primary complete | 804,54(51.53-56.66) | 682,45.9(43.33-48.46) |
|  | Secondary complete | 567,66.9(63.73-70.18) | 279,33(29.81-36.26) |
|  | Higher | 322,77.6(73.46-81.69) | 92,22.3(18.30-26.53) |
| Working Status | Never worked | 82,4.46(.0357-.0552) | 1750,95.5(94.47-96.42) |
|  | Currently Working | 920,64.3(61.79-66.82) | 510,35.6(33.17-38.20) |
|  | Currently not Working | 1476,54.3(52.44-56.23) | 1240,45.6(43.76-47.55) |
| Partner | With Partner | 1979,57.2(55.56-58.88) | 1479,42.7(41.11-44.43) |
|  | Without Partner | 498,19.7(18.21-21.36) | 2022,80.2(78.59-81.74) |
| Wealth Index | Most deprived | 532,40(37.38-42.72) | 797,59.9(57.27-62.61) |
|  | 2 | 527,39.8(37.21-42.56) | 795,60.1(57.43-62.78) |
|  | 3 | 450,38.50(35.72-41.38) | 718,61.5(58.61-64.27) |
|  | 4 | 516,43.1(40.31-46.00) | 680,56.8(53.99-59.68) |
|  | Most affluent | 452,46.8(43.65-50.04) | 513,53.1(49.95-56.34) |
| Region | East | 584.9,43.4(40.73-46.09) | 762.8,56.6(53.83-59.19) |
|  | West | 498,38.5(35.82-41.19) | 796,61.4(58.72-64.09) |
|  | North | 129,40.5(35.23-46.32) | 188,59.4(53.67-64.76) |
|  | South | 486,38.3(35.64-41.06) | 782,61.67(58.93-64.35) |
|  | North-east | 48,38.7(30.35-48.23) | 75,61.23(51.76-69.64) |
|  | Central | 731,44.8(42.41-47.29) | 899,55.1(52.70-57.58) |

Supplementary Table S3: Variance Inflation Factor for regression model

| Variables | VIF |
| --- | --- |
| Age Groups | 1.12 |
| Gender | 1.58 |
| Residence | 1.39 |
| Partner | 1.69 |
| Caste | 1.12 |
| Education | 1.37 |
| Working Status | 1.25 |
| Wealth Index | 1.09 |
| Region1 | 1.01 |
| Mean VIF | 1.29 |
